# A high-throughput seed germination assay for root parasitic plants

**DOI:** 10.1186/1746-4811-9-32

**Published:** 2013-08-06

**Authors:** Jean-Bernard Pouvreau, Zachary Gaudin, Bathilde Auger, Marc-Marie Lechat, Mathieu Gauthier, Philippe Delavault, Philippe Simier

**Affiliations:** 1 Laboratoire de Biologie et de Pathologie Végétales EA 1157, SFR 4207 QUASAV, Nantes University, 44322 Nantes, France

**Keywords:** Broomrape, Germination, MTT, Parasitic plants, *Phelipanche ramosa*, Strigolactone

## Abstract

**Background:**

Some root-parasitic plants belonging to the *Orobanche*, *Phelipanche* or *Striga* genus represent one of the most destructive and intractable weed problems to agricultural production in both developed and developing countries. Compared with most of the other weeds, parasitic weeds are difficult to control by conventional methods because of their life style. The main difficulties that currently limit the development of successful control methods are the ability of the parasite to produce a tremendous number of tiny seeds that may remain viable in the soil for more than 15 years. Seed germination requires induction by stimulants present in root exudates of host plants. Researches performed on these minute seeds are until now tedious and time-consuming because germination rate is usually evaluated in Petri-dish by counting germinated seeds under a binocular microscope.

**Results:**

We developed an easy and fast method for germination rate determination based on a standardized 96-well plate test coupled with spectrophotometric reading of tetrazolium salt (MTT) reduction. We adapted the Mosmann’s protocol for cell cultures to germinating seeds and determined the conditions of seed stimulation and germination, MTT staining and formazan salt solubilization required to obtain a linear relationship between absorbance and germination rate. Dose–response analyses were presented as applications of interest for assessing half maximal effective or inhibitory concentrations of germination stimulants (strigolactones) or inhibitors (ABA), respectively, using four parameter logistic curves.

**Conclusion:**

The developed MTT system is simple and accurate. It yields reproducible results for germination bioassays of parasitic plant seeds. This method is adapted to high-throughput screenings of allelochemicals (stimulants, inhibitors) or biological extracts on parasitic plant seed germination, and strengthens the investigations of distinctive features of parasitic plant germination.

## Background

Most of the root parasitic plants grow in natural habitats in equilibrium with their host plants all-over the world. In contrast, few species adapted to a different way of living as weeds in human-made ecosystems, and cause severe damage in major crops, such as some *Orobanche* and *Phelipanche* species (broomrapes) in the Mediterranean basin in addition to some *Striga* species in Africa and Asia [1]. Control of these root parasitic weeds is difficult and an integrated crop management is recommended to reduce the infestations using a rational combination of cultural, chemical, biological and genetic methods of control [2]. However, a biological trait of this kind of weeds make complex any crop management: each plant can produce an important amount of tiny seeds showing long viability in the soil.

Seed germination is a key component of pathogenicity of obligate parasitic weeds. This step is controlled allelochemically in the rhizosphere. After a short conditioning period under sufficient warm and wet conditions, seed germination is triggered by stimulant molecules which are released by the roots of neighboring host plants. Many secondary metabolites were identified as germination stimulants. Most of them corresponds to strigolactones (SLs) [3] but isothiocyanates [4,5], dehydrocostus lactone [6], peagol, peagoldione [7], chalcones, peapolyphenols [8], soyasapogenol B and trans-22-dehydrocampesterol [9] have been also identified as stimulants. It was recently demonstrated that seeds of *P*. *ramosa* needs a minimal period of conditioning before that stimulant GR24 (a synthetic SL) could trigger germination by breaking ABA dormancy [10]. Inversely, broomrape seed germination can be prevented in the rhizosphere by inhibitors including trigoxazonane present in root exudates of the allelopathic plant *Trigonella*[11], several trichotecenes produced by the potential biocontrol fungi agents, *Myrothecium verrucaria* and *Fusarium compactum*[12], 7-hydroxylated simple coumarins and both naringenin and gallic acid present in root exudates of resistant sunflower and pea, respectively [13-15]. In this context, many investigations are conducted on the identification and the characterization of the germination stimulants (e.g. [16-18]). Fundamentally, their interest corresponds to a better understanding of the plant-plant interaction. There is also a great agronomical challenge to control these parasitic weeds, either by preventing seed germination through biocontrol agents or intercropping with allelopathic plants, or in contrast by promoting seed germination in the absence of host plants by cropping false hosts in order to reduce the seed bank of the soils [2].

**Figure 1:**
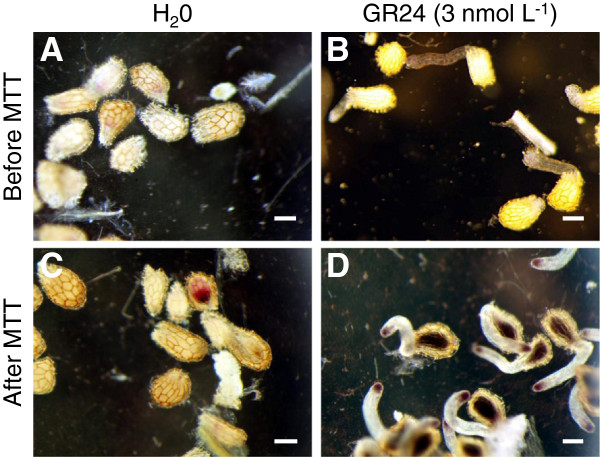
**Reduction of MTT by conditioned and germinated seeds of *P. ramosa.*** Control, conditioned seeds incubated without GR24 **(A, C)** and assays 7 days after stimulation with GR24 (3 nmol L^-1^) **(B**, **D)**. Photographs were taken before **(A, B)** and after **(C, D)** incubation with MMT for 6 hours. Bars 100 μm.

All these works need a rapid and reliable bioassay of seed germination allowing a high-throughput screening of molecules, root exudates or plant extracts. Until now, germination rate is usually evaluated in Petri-dish by counting germinated seeds under a binocular microscope. This method is time-consuming, tedious due to the minute size of seeds (about 200 μm in diameter, Figure 1), and inappropriate if hundred of samples have to be analyzed. A miniaturization assay in 96-well plates is available for the screening of numerous *Arabidopsis* seedling accessions towards *P*. *ramosa* and *P*. *aegyptiaca* seed germination, and uses 10–50 seeds in water per well in which seedlings are transferred [19]. This method remains tedious notably due to the germination rate determination by binocular microscopy. We propose in this paper to substitute this step by a spectrophotometric determination of germination rate. Indeed to make easier and faster measurements for high-throughput studies, we developed a standardized 96-wells plate germination test coupled with spectrophotometric reading of methylthiazolyldiphenyl-tetrazolium bromide (MTT) reduction [20]. The analog staining method using the tetrazolium salt 2,3,5 triphenyl tetrazolium chloride (TTC) demonstrated early changes in metabolic activity in *O*. *crenata* and *P*. *ramosa* seeds during germination [21,22]. Indeed TTC staining of seeds stimulated with GR24 for 5 min displayed an increase in metabolic activity 30 h after the stimulation. The radicle was observed after 40 h in seeds and germination was achieved 3 days after treatment (root protrusion) [21]. The tetrazolium salt systems are a mean of measuring the activity of living cells thanks to mitochondrial dehydrogenase activities [20]. The proposed MTT method is simple and accurate, and yields reproducible results. We demonstrate here that the MTT method can be adapted for high-throughput germination bioassays, then giving an efficient tool for detailed investigations on seed germination, as presented for example in this paper as a proof of concept with data on the effects of ABA level on *P*. *ramosa* seed germination.

## Results

The Mossman test use MTT as a marker of metabolic activities. When dissolved in a medium, oxidized MTT is yellowish in color. When applied to biological materials, the yellow MTT is reduced to purple formazan crystals by mitochondrial enzymatic activities. These crystals are insoluble in aqueous solutions, they are dissolved in acidified isopropanol and the resulting purple solution can be then measured spectrophotometrically. Following extrapolation, the results of this assay can be expressed in term of number of viable cells. Cytotoxicity of tested molecules or biological extracts can be then routinely assessed.

**Figure 2:**
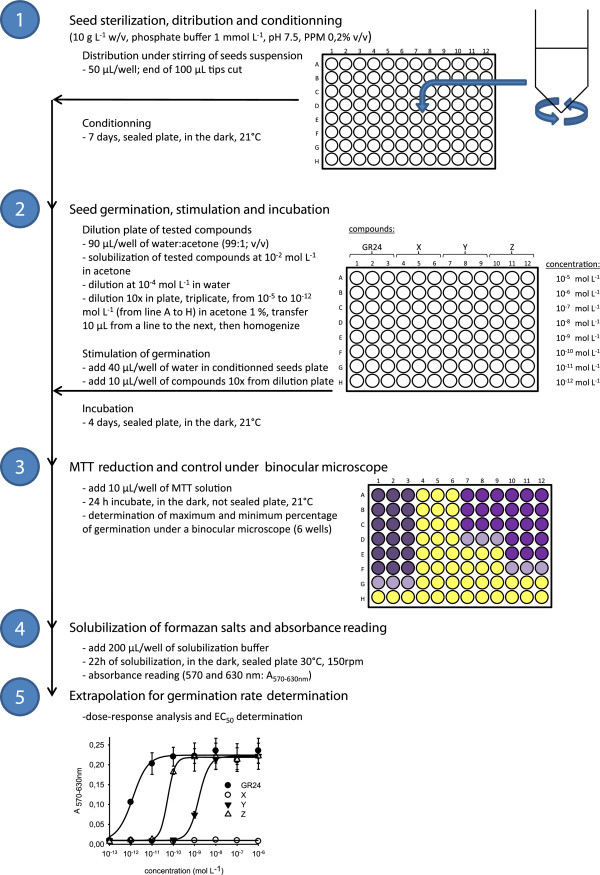
**Experimental design for the proposed high**-**throughput seed germination assay for root parasitic plants.**

In the context of our study, when MTT was added 7 days post GR24-stimulation (dps), germinated broomrape seeds reduced MTT into formazan mainly at the apex of the radicle and in the seed body (Figure 1B, D). This activity was not observed in the non-germinated seeds (Figure 1A, C). Some of them displayed a red deposition in the seed body that did not correspond to the purple deposition of formazan salts (Figure 1C). First, the observed differential staining between germinated seeds and non-germinated seeds facilitated the observation of the germination process under a binocular microscope. Secondly, produced formazan crystals could be solubilized for absorbance reading. Therefore the Mossman’s test has been adapted and validated in order to develop high-throughput bioassays for germination of obligate parasitic plant seeds. This method could be divided in five steps summarized in Figure 2: 1) seed sterilization, seed distribution and conditioning in 96 well plates, 2) seed germination stimulation and incubation, 3) MTT reduction and visual control under a binocular microscope, 4) solubilization of formazan salts and absorbance reading and 5) extrapolation of absorbance values for determination of germination rate. First of all, some steps had to be optimized including the pH buffer for seed germination and the length of seed germination after GR24-addition, MTT incubation, and formazan salt solubilization, as detailed here below.

### Optimum pH for seed germination

**Figure 3:**
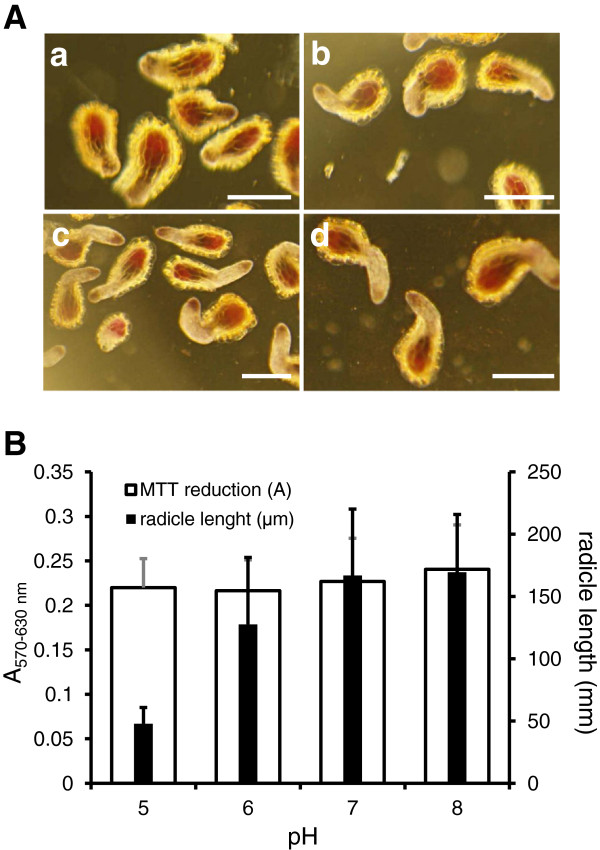
**pH impact on seed germination and radicle growth in *Phelipanche ramosa.*** Conditionned seeds were stimulated with GR24 (3 nmol L^-1^) then incubated 7 days in Na, K phosphate medium (1 mmol L^-1^) at pH 5 **(a)**, 6 **(b)**, 7 **(c)** and 8 **(d)**. **A**- photographs were taken after MTT incubation for 6 hours. Scale bars 200 μm. **B**- pH impact on MTT reduction by absorbance measurement at 530 nm minus at absorbance at 630 nm (± confidence intervals α = 0.05, n = 3), radicle length (± confidence intervals α = 0.05, n = 400), germination percentage were 95% ±4%.

Phosphate buffer in the germination medium were tested at pH 5, 6, 7 and 8. MTT was added 7-dps. When assessed under binocular microscope, the germination percentage was not affected by pH and reached 95 ± 5% (Figure 3). Similarly, following formazan crystal solubilization, absorbance was not significantly different (SNK, p < 0.05). However, when radicle was observed, a strong reduction in length was observed for acid pH values (Figure 3). A pH of 7.5 which did not impact germination and radicle development was chosen for MTT assay.

### Kinetics of germination and MTT staining

**Figure 4:**
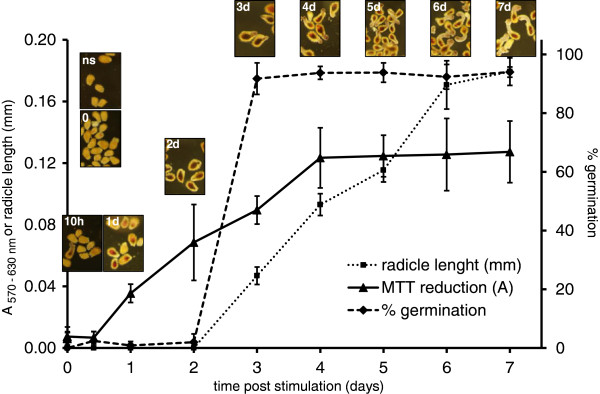
**Kinetics of seed germination and radicle growth in *Phelipanche ramosa. ***Conditionned seeds of *P*. *ramosa* were stimulated or not (ns) with GR24 3 nmol L^-1^ for 0, 10 hours, 1, 2, 3, 4, 5 , 6 and 7 days, then incubated with the MTT for 6 hours. Scale bars = 200 μm. Kinetics of MTT reduction was followed by absorbance measurement at 530 nm minus absorbance at 630 nm (A_570-630nm_), radicle length and germination percentage counting under binocular microscope (± confidence intervals α = 0.05, n = 8).

The kinetics of germination rate, radicle length and MTT reduction were followed concomitantly during 7 dps in 96 well plate (≈125 seeds per well) (Figure 4). GR24 applied at 3 nmol L^-1^ induced 93 ± 4% of germination 7 dps (Figure 4). No staining was observed during the first ten hours (Figure 4 0-10h) except for a few spontaneously germinated seeds (2 ± 3%, data not shown). The slight red deposition appeared in seed body at 1 dps while the purple staining marked the radicle area (Figure 4 1d). Then MTT reduction was spread throughout the seed (Figure 4 2d). Radicle protrusion was observed 3 dps when a small germ tube (47 ± 6 μm) was measured (Figure 4 3d). Seed germination was synchronized (92 ± 5%, ±EC, n =8 wells). No significant change in germination rate was observed 3 dps (ANOVA, SNK, α = 0.05). The radicle elongated until 6 dps when it reached its maximum size (176 ± 16 μm) (Figure 4 4d-7d). Therefore *sensus stricto* germination occurred in the first 3 dps and the following 3 days consisted in post-germination development. A significant reduction of MTT could be detected spectrophotometrically from 1 dps (ANOVA, SNK, α = 0.05). The activity increased regularly until 4 dps and then kept a maximal and stable value. In accordance with the kinetics obtained in these experimental conditions (21°C, pH 7.5), 4 dps duration was chosen for the MTT assay as an appropriate timing for maximal germination rate and MTT reduction.

### Length of MTT incubation

The reduction of MTT was observed 4 dps only at the apex of the radicle after two hours of incubation in the MTT medium (Additional file 1A), while it was spread throughout the germinated seeds over 6h of incubation. As shown by absorbance determination, once formazan was solubilized MTT reduction in germinated seeds was optimal for a 24 h-incubation in the MTT medium (Additional file 1B; ANOVA, SNK, α = 0.05). Therefore, in accordance with the kinetics, 24 h-incubation in the MTT medium of the germinated seeds (4 dps) was chosen for the MTT assay.

### Length of formazan salt solubilization

Using the Mossman procedure without modifications, absorbance was low few minutes after addition of solubilization buffer since most of the formazan salt deposition remained in the seed body. Germinated seeds are structurally more complex than cells due to the presence of teguments and others tissues, then the solubilization step needed to be extended. First, formazan salt solubilization from seeds was followed by absorbance measurement during incubation in culture chamber conditions without agitation (21°C, darkness, Additional file 2 Maximum absorbance was obtained after 40 h of incubation and remained stable during 24 h (Additional file 2). Complete solubilization of formazan from seeds at this time point was confirmed through microscope observations (data not shown). The duration of solubilization could be significantly reduced by incubating the plates at 30°C in darkness under orbital shaking (150 rpm). Less than 22 h was required in those conditions for complete formazan solubilization and the absorbance was also stable during one day. These conditions (30°C, 22 h, under shaking) were selected for the MTT assay.

### Linear relationship between MTT reduction and germination rate

**Figure 5:**
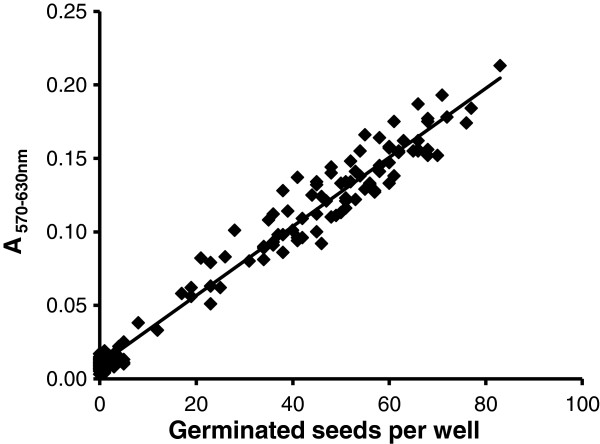
**Linear relationship between MTT reduction and germination rate for *P. ramosa *seeds.** y = 0.0023 x + 0.01; R^2^ = 0.976, measurements 4 days post GR24 stimulation.

To prove that the test can be used quantitatively to estimate germination rate, germination assays in 96-well plates were prepared using *P*. *ramosa* conditioned seeds and various GR24 concentrations from 10^-15^ to 10^-6^ mol L^-1^. Controls were done without GR24 and with GR24 at used concentrations without conditioned seeds. MTT solution was added in each well 4 dps. Following 24 h-incubation in MTT solution, plates were transferred at 30°C in darkness under orbital shaking (150 rpm) during 24 h for complete formazan salt solubilization. No MTT reduction was observed in the control wells containing only GR24 solutions (data not shown), proving that GR24 did not interact with MTT through oxidoreduction processes. As observed in Figure 5 and Table 1, the relationship between germinated seeds per well or germination percentage and absorbance was linear (R^2^ =0.976 and R^2^ =0.891, respectively). Therefore absorbance could be converted into germination efficiency expressed as either germinated seed number per well or germination percentage.

**Table 1:** Linear relationship between MTT reduction and seed germination rate for various parasitic plants

***Species***	**Lot**	**Seeds/well**	**Linear regression**	**R**^**2**^
*Phelipanche ramosa*	St Martin de Fraigneau, France, 2005	120	A = 0.0023 gs + 0.011	0.976
*Orobanche cumana*	Longeville-sur-mer, France, 2009	110	A = 0.0019 gs + 0.009	0.965
*Orobanche minor*	Japan, 2010	80	A = 0.0043 gs + 0.032	0.977
*Striga hermonthica*	Gadarif, Soudan, 1999	90	A = 0.0127 gs + 0.016	0.920

A, Absorbance; gs, number of germinated seeds per well.

Using similar conditions and time courses, linear relationship between absorbance and germination efficiency was demonstrated for seeds of three other parasitic plants, *O*. *minor*, *O*. *cumana* and *S*. *hermonthica* (Table 1), despite difference in germination velocity could be observed according to the species: *O*. *cumana* and *S*. *hermonthica* seeds germinated and developed a long radicle (length > 200 μm) 4 dps, while no radicle emergence from micropyle was observed from germinated *O*. *minor* seeds at this time point.

### Technical tips and tricks

Seed distribution in plate is the main critical step of the proposed protocol. Because the signal is proportional to the germinated seed number per well, seed distribution must be homogenous between wells in order to valid linear relationship between absorbance and germination percentage. So the suspension of sterilized seeds must be maintained in the tube under stirring throughout seed distribution in plate using a pipette. The end of tips must be cut to avoid seed clogging and mismatch seed distribution. Seed number per well should be both sufficient to neglect low variation of seed number per well and not too high for absorbance reading and control under microscope. Using the described conditions, 125 ± 11 seeds are usually added per well (± SD, n = 96). For seeds difficult to maintain in suspension, as observed in this study for *O*. *cumana* and *O*. *minor*, addition of Silwet-L77 or Triton X-100 as a detergent in the suspension medium facilitated seed distribution in plate. Concentration should be less than 0.001% otherwise higher concentrations inhibited germination (data not shown).

Most of the tested compounds were dissolved in solvents such as acetonitrile, acetone, ethanol, or dimethylsulfoxide. Acetone, acetonitrile or ethanol percentages less than 0.1% did not alter the response of *P*. *ramosa* seed to GR24 (EC50 and maximum germination percentage). Dimethylsulfoxide can be used up to 1% (data not shown).

Compatibility of different buffers with seed germination was checked. Sodium phosphate buffer was compatible up to 10 mmol L^-1^ while sodium-potassium phosphate and HEPES buffers can be used up to 20 mmol L^-1^. Higher concentrations dramatically inhibited *P*. *ramosa* seed germination (data not shown). Therefore Hepes or phosphate buffer were used routinely from 1 to 5 mmol L^-1^ at pH 7.5.

Despite seed sterilization and precautions during experiments, MTT reduction could be performed by contaminant microbial dehydrogenases in wells, then generating noise or false positives. To overcome this risk, Plant Preservative Mixture^TM^ (PPM), a heat-stable broad spectrum biocide [23], has been added to the Na, K phosphate buffer before seed suspension and distribution in each well. PPM concentrations from 0.01 to 0.1% did not affect the dose–response curve of GR24 while concentrations above 1% inhibited *P*. *ramosa* seed germination (data not shown). PPM was usually used at 0.1% for the MTT assay.

### Examples of application as proofs of concept

#### Interest for dose–response analysis

**Figure 6:**
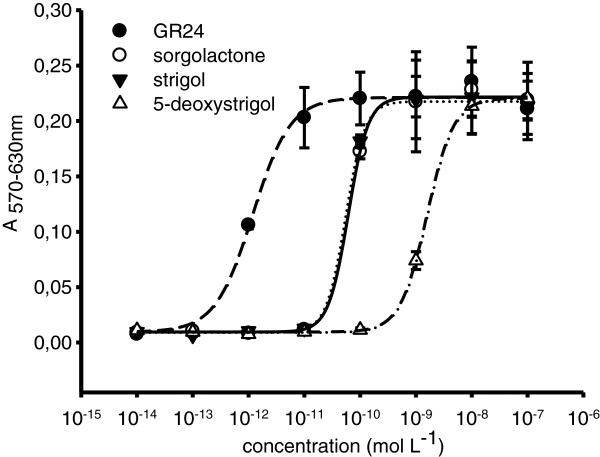
**Activities of strigolactones on *P. ramosa *seed germination.** Dose response activities of sorgolactone, strigol, 5-deoxystrigol and GR24 are modeled using a Four Parameter Logistic Curve (sorgolactone, solid line; strigol, dot line; 5-deoxystrigol, dash-dot line and GR24, dash line).

Standard MTT assay in 96-well plate was checked for dose–response analysis with *P*. *ramosa* seeds exposed to a large range of concentrations of candidate compounds (Figure 6). Using only one 96-well plate in this example, dose–response curves were obtained, with dilution replicates, for three natural SL and GR24 between 10^-14^ and 10^-7^ mol L^-1^. Half maximal effective concentration EC_50_ and maximum of germination percentage of each compound were determined using a Four Parameter Logistic Curve (Table 2). EC_50_ reflects both the stimulant affinity to the receptor and the effectiveness of the induced response. In this example, since the four SLs triggered high and similar values of maximum germination (ANOVA, P <0.05), EC_50_ can be discussed as a specific indicator of SL affinity to the receptor. So GR24 displayed the highest affinity, with EC_50_ at the picomolar range as also reported in previous studies for this *P*. *ramosa* genotype [4,24]. Sorgolactone and strigol affinities were about 50-fold lower while 5-deoxystrigol affinity was about 1000-fold lower (EC_50_ at the nanomolar range). This example proves that the MTT assay is appropriate for rapid and robust comparison of stimulant activities and will make easier structure-activity relationship studies [25-27].

**Table 2:** **Activity of different strigolactones on *P***. ***ramosa *seed germination**

	**EC**_**50 **_**(mol L**^**-1**^ **± SE)**	**A max (± SE)**	**R**^**2**^
Sorgolactone	6.1 ± 0.9 10^-11^	0.222 ± 0.003	0.999
Strigol	5.4 ± 0.8 10^-11^	0.218 ± 0.002	0.999
5-deoxystrigol	1.6 ± 0.05 10^-9^	0.221 ± 0.002	1.000
GR24	1.4 ± 0.4 10^-12^	0.222 ± 0.007	0.989

Analyses were performed with a Four Parameter Logistic Curve (maximum of absorbance, Amax).

#### Interest for phenotypic studies

**Figure 7:**
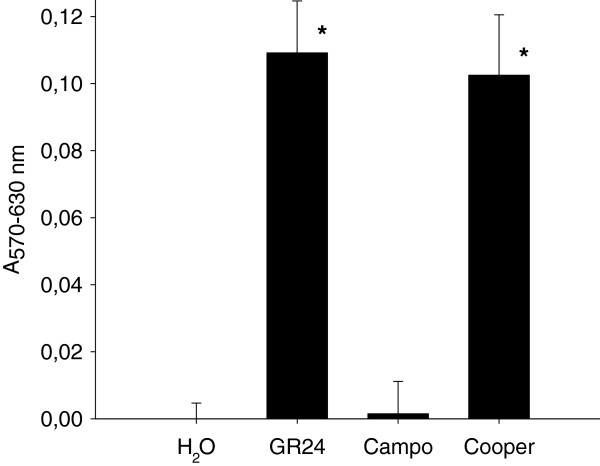
**Root exudates activity of two *Brassica napus *elite lines on *P. ramosa *seed germination.** Negative control, H_2_0; synthetic stimulant, GR24 3 nmol L^-1^ (means ± confidence intervals, α = 0.05, n = 6). Bars with asterisk are significantly different from the negative control (Holm-Sidak method, P < 0.05).

Standard MTT assay in 96-well plate was checked for assessing susceptibility of seeds from four parasitic plants to GR24 (Table 3). One dilution plate of GR24 in triplicate from 10^-6^ to 10^-13^ mol L^-1^ was used. In this example, *P*. *ramosa* seeds displayed the highest sensitivity to GR24 at the picomolar range while *O*. *cumana* and *O*. *minor* seeds displayed EC values at the nanomolar range. Those findings support the hypothesis of variability and evolution of SLs receptors in parasitic plants [24-26], and prove the MTT assay is appropriate for discriminating different parasitic plant species or genotypes or races according to their sensitivity to germination stimulants. Moreover, the MTT assay is also adapted to evaluate the stimulant activity of plant root exudates on parasitic plant seed germination. As a proof of concept, Figure 7 shows the divergent activity of two oilseed rape lines on *P*. *ramosa* seed germination.

**Table 3:** GR24 activity on seed germination in various parasitic plants

	**EC**_**50 **_**(mol L**^**-1**^ **± SE)**	**% max (± SE)**	**R**^**2**^
*Phelipanche ramosa*	1.4 ± 0.4 10^-12^	89 ± 3	0.999
*Orobanche cumana*	4.0 ± 1.1 10^-9^	80 ± 3	0.976
*Orobanche minor*	1.2 ± 0.3 10^-8^	83 ± 4	0.936
*Striga hermonthica*	2.4 ± 0.7 10^-11^	54 ± 2	0.912

Analyses were performed with a Four Parameter Logistic Curve (maximum of germination percentage, % max).

#### Interest for physiological studies

GR24 stimulates *P*. *ramosa* seed germination by breaking ABA dormancy [10]. Experiments using two 96-well plates with various ABA and GR24 concentrations proved that ABA inhibits germination in the presence of GR24 in a dose dependant manner (Table 4A). ABA IC_50_ ranged between 1.0 and 2.3 10^-6^ mol L^-1^, without significant difference in the GR24 concentrations range tested (SNK, p < 0.05). Using ABA concentrations lower than ABA IC_50_, no significant difference was obtained for maximum absorbance or maximum germination percentage with a large range of GR24 concentrations between 1 10^-8^ and 5 10^-12^ mol L^-1^ (SNK, p < 0.05). In the same way, ABA did not affect GR24 EC_50_ (SNK, p < 0.05; Table 4B). Nevertheless ABA reduced significantly maximum germination percentage for GR24 concentration higher than 10^-7^ mol L^-1^(SNK, p < 0.05). These findings prove that ABA-related inhibition and GR24-related stimulation on *P*. *ramosa* seed germination are antagonist and uncompetitive processes. Thus standard MTT assay in 96-well plate is also an appropriate tool to study the physiological aspects of seed germination in parasitic plants.

**Table 4:** **Antagonistic role of ABA versus GR24 activities on seed germination in *P***. ***ramosa***

**A**			
**GR24 (mol L**^**-1**^**)**	**IC**_**50 **_**(mol L**^**-1**^ **± SE)**	**A max (± SE)**	**% max (± SE)**
1 10^-8^	2.3 10^-6^ ± 6 10^-7^	0.332 ± 0.009	81 ± 0.3
1 10^-9^	1.4 10^-6^ ± 1.4 10^-7^	0.377 ± 0.004	92 ± 1.5
1 10^-10^	1.2 10^-6^ ± 2.8 10^-7^	0.395 ± 0.013	94 ± 0.8
5 10^-11^	1.4 10^-6^ ± 4.2 10^-7^	0.387 ± 0.013	94 ± 0.8
1 10^-11^	1.6 10^-6^ ± 1.1 10^-7^	0.394 ± 0.014	96 ± 1.1
5 10^-12^	1.0 10^-6^ ± 1.1 10^-7^	0.366 ± 0.013	89 ± 0.6
1 10^-12^	nd	0.024 ± 0.12	4 ± 0.4
**B**			
**ABA (mol L**^**-1**^**)**	**EC50 (mol L**^**-1**^**,** **± SE)**	**A max (± SE)**	**% max (± SE)**
5 10^-5^	nd	0.004 ± 0.002	2 ± 2
1 10^-5^	nd	0.004 ± 0.002	1 ± 2
5 10^-6^	2.8 10^-12^ ± 1.2 10^-12^	0.070 ± 0.020	15 ± 2
1 10^-6^	4.8 10^-12^ ± 3.1 10^-12^	0.260 ± 0.010	62 ± 1
5 10^-7^	3.1 10^-12^ ± 2.1 10^-12^	0.330 ± 0.010	80 ± 3
1 10^-7^	4.8 10^-12^ ± 2.5 10^-12^	0.370 ± 0.010	89 ± 1
5 10^-8^	4.8 10^-12^ ± 2.5 10^-12^	0.370 ± 0.010	89 ± 1
1 10^-8^	4.6 10^-12^ ± 8.3 10^-12^	0.400 ± 0.010	95 ± 1
5 10^-9^	4.5 10^-12^ ± 7.7 10^-12^	0.400 ± 0.010	96 ± 1
1 10^-9^	4.6 10^-12^ ± 2.6 10^-12^	0.400 ± 0.020	98 ± 2
1 10^-10^	1.3 10^-12^ ± 3.6 10^-12^	0.390 ± 0.020	94 ± 2
0	3.0 10^-12^ ± 0.7 10^-12^	0.350 ± 0.010	85 ± 1

**A**- ABA IC_50_ versus GR24 concentration. **B**- GR24 EC_50_ versus ABA concentration (maximum of absorbance, Amax; maximum of germination percentage, % max; not determined, nd).

## Discussion

Using a microscope, the germination rate of the tiny seeds of parasitic plants is usually determined by counting seeds showing a protruded radicle through the seed coat [8,28-32]. As reported for other dyes such as TTC [21,22], blue ink or Coomassie Blue [33], MTT staining contrasts the germinated seeds, and then facilitates observation and counting. On the other hand, unlike those colorants, MTT staining is appropriate for a spectrophotometric reading. So we described here a standard MTT assay in plate for the spectrophotometric determination of germination rates of parasitic plant seeds. It was carried out according to the Mossman‘s procedure for cell cultures [20] with major modifications due to the structural complexity of seeds compared to cells.

The kinetics of MTT staining during seed germination facilitates the discrimination between the induction of germination that was characterized by purple staining due to metabolic activities of the embryo, and the radicle protrusion and elongation (Figure 4). As shown for *P*. *ramosa* seeds (Figure 3), germination was induced by GR24 under a large range of pH while radicle protrusion and elongation were strongly affected by acid pH values. Within the MTT assay, pH effects on radicle length did not impact the absorbance reading since absorbance was correlated to germination rate independently to radicle elongation (Figures 3 and 5). Within the classical method using a microscope (without MTT), the distinction between non germinated seeds and germinated seeds with a very short protruded radicle (= false negative) is very difficult, increasing the risk of germination rate underestimation. The proposed spectrophotometric MTT assay gives a significant advantage for preventing these false negatives. However, because this method is not suitable to discern effects on radicle elongation, it is not relevant for testing compounds that affect radicle elongation.

XTT (2,3-bis-(2-methoxy-4-nitro-5-sulfophenyl)-2H-tetrazolium-5-carboxanilide) or other tetrazolium salts that form water-soluble formazan has been proposed to replace MTT in the Mossman’s procedure [34], yielding higher sensitivity and a higher dynamic range. In addition, the formed formazan dye is water soluble, then avoiding the final solubilization step. While first attempts to replace MTT by XTT in the incubation medium of *P*. *ramosa* seeds were positive (data not shown), experiments for XTT assay standardization were not carried out. Indeed, the fact that XTT does not contrast seeds represents a major inconvenient for the optical control of seed germination in plate.

Conditioning and germination of parasitic plants seeds could be affected by environmental conditions as temperature, light, water potential or salinity [35-38]. For *P*. *ramosa*, the developed method has shown that seeds germinated over a large range of pH (5 to 8). This finding proves that the spectrophotometric MTT assay is adapted to high throughput studies of environmental factors on kinetics of parasitic plant seed germination. The MTT assay is also appropriate for a number of other (high throughput) studies on parasitic plants including structure-activity relationship analysis of germination stimulants or inhibitors (allelochemicals), bioguided purification of natural allelochemicals from plant extracts, and studies on seed response to allelochemicals through pharmacological approaches. The MTT assay is also benefit for screening a number of plant root exudates (Figure 7) as part of studies on host spectrum of parasitic plants, screenings of germplasms for resistance breeding, and applied researches on allelopathic plants acting as potential catch or false host crops in order to reduce the parasitic pressure in infested fields.

## Conclusions

To make easier and faster measurements of germination rate of parasitic plant seeds for high-throughput studies, we developed a spectrophotometric and standardized 96-well plate assay. The assay is based on the relationship between the induction of seed germination by stimulants and the increase in dehydrogenase activities of germinating seeds that is determined by reduction of exogenous methylthiazolyldiphenyl-tetrazolium bromide (MTT). Compare to the classical microscope assay, the developed MTT assay decreases the length of experiments and especially the duration of the reading step (Table 5). In addition, several examples of applications were also shown, attesting that this assay will be a useful tool for both fundamental and applied researches on plant-parasitic plant interactions.

**Table 5:** Advantages and disadvantages of germination determination methods

	**Classical microscope assay**	**Spectrophotometric MTT assay**
Duration of the experiment	7 days	6 days
Duration of the reading step (96 well plate)	2-3 h	< 5 min
Sensitivity to false negative	yes	no
Radicle elongation determination	yes	no

## Methods

### Plant material

Seeds of *Phelipanche ramosa* L. Pomel (genetic type 1, [39]) were collected in 2005 from Saint Martin de Fraigneau, France, on broomrape-parasitized winter oilseed rape (*Brassica napus* L.). Seeds of *Orobanche cumana* Wallr. were collected in 2009 from Longeville-sur-mer, France, on broomrape parasitizing sunflower (*Helianthus annuus* L.); seeds of *Orobanche minor* Sm were kindly provided by Pr K. Yoneyama (Japan) and seeds of *Striga hermonthica* (Del.) Benth were collected in 1999 from Gadarif, East Sudan. The seeds were stored dry in the dark at 25°C until use.

Seeds of *Brassica napus* (L.) elite lines, Campo and Cooper, were provided by the breeder companies, Dekalb and Advanta respectively. Plants were grown and root exudates were collected 7 weeks after sowing according to Labrousse *et al*. (2001) [40].

### Chemicals

GR24, sorgolactone, strigol and 5-deoxystrigol were kindly provided by Dr F-D. Boyer [24]. ABA and MTT (3-[4,5-dimethylthiazol-2-yl]-2,5-diphenyltetrazolium bromide) were purchased from Sigma Aldrich (St Louis, MO, USA). They were resuspended in acetone at 10 mmol L^-1^, then diluted with water at 1 mmol L^-1^ (water/acetone; v/v; 99/1). Control was prepared as acetone 1% (v/v). Dilutions of 1 10^-3^ mol L^-1^ to 1 10^-15^ mol L^-1^ were then performed in water/acetone (v/v; 99/1). ABA was solubilized in basified water at 10 mmol L^-1^ and then diluted in phosphate buffer (1 mmol L^-1^, pH 7.5) from 10^-3^ to 10^-10^ mol L^-1^. MTT was prepared at 5 g L^-1^ in distilled water then filtered at 0.2 μm (RC filters, Millipore) and stored at 4°C in the dark.

### MTT assays

#### Seed sterilization, distribution and conditioning in 96 well plates

Seeds were surface-sterilized according to Vieira Dos Santos *et al*. (2003) [41] with some minor modifications. Seeds were surface-sterilized in vigorous agitation in a 9.2° sodium hypochlorite solution for 5 min. Hypochlorite was removed by washing three times 30 s then three times 5 min with autoclaved deionised water. Then seeds were resuspended at a density of 10 g L^-1^ (dry seed weight/v) in Na, K phosphate buffer (1 mol L^-1^, pH 7.5) containing PPM 0.2% (Plant Preservative Mixture, Kalys, Bernin, France), and distributed under stirring in 96-well plates (Cell Culture Multiwell Plate Cellstar; Greiner Bio-One, Frickenhausen, Germany), around 50 μL ≈ 125 seeds per well. Seeds were conditioned in sealed plates during 7 days at 21°C in darkness, excepted *S hermonthica* seeds that were conditioned at 30°C.

#### Seed germination, stimulation and incubation

Fresh root exudates (0.2 μm filtered), GR24, ABA or control were added and volumes were adjusted to 100 μL with water. Phosphate buffer was therefore adjusted at 500 μmol L^-1^ (pH 7.5) and acetone at 1/1000 (v/v). Negative controls were made with solvents, buffer and seeds minus tested chemicals. Blanks containing complete assay without seeds were also included in each test. Each molecule and concentration was tested on 3 well replicates against 3 replicates of blanks. Plates were incubated for seed germination as previously described at 21°C or 30°C according to the species.

#### MTT reduction and control under a binocular microscope

The MTT assays were carried out according to Mossman (1983) [20] with modifications. Ten micro litters of MTT solution per well was added. Plates were replaced in culture chamber for 24 hours in darkness or overnight (unsealed plates, at 21 or 30°C according to the species). As a control of seed germination and MTT staining, both germinated seeds showing radicle protrusion and non-germinated seeds were counted before and after MTT addition and incubation using a zoom stereo microscope with 0.5 and 2 x objectives with darkfield illumination (SZX10, Olympus). Images were captured with a digital camera (E-330, Olympus) controlled by cell-A software (Olympus, Japan).

#### Solubilization of formazan salts and absorbance reading

Two hundred microlitters of solubilization buffer (Triton X-100 10%, HCl 0.04 mol L^-1^ in isopropanol) per well were added. Formazan salt deposition was solubilized in the lysis buffer for 22 hours in orbital shaker (150 rpm, 30°C). Absorbance was read using a *EL800* Absorbance Microplate Reader (Biotek, Winooski, United States) equipped with 570 nm and 630 nm filters. For each well, the absorbance at a reference wavelength of 630 nm was subtracted from a test absorbance of 570 nm (A_570-630nm_).

#### Extrapolation of absorbance values for germination rate determination and dose response curves analysis

Linear regression, absorbance as a function of germination percentage, was computed with SigmaPlot 10.0. Dose–response curves, absorbance or germination percentage as a function of GR24 or ABA concentration, were modeled with a four parameter logistic curve computed with SigmaPlot 10.0.

### Statistical analysis

Data were subjected to analysis of variance (ANOVA) with EC_50_, IC_50_ or germination percentage as the factor. Germination percentages were previously sin^-1^ (square root)-transformed to normalize the ratios.

## Abbreviations

A: Absorbance; ABA: Abscissic acid; Dps: Day post stimulation; EC50: Half maximal effective concentration; IC50: Half maximal inhibitory concentration; MTT: Methylthiazolyldiphenyl-tetrazolium bromide; PPM: Plant Preservative Mixture; SL: Strigolactones; TTC: 2,3,5 Triphenyl Tetrazolium chloride; XTT: 2,3-bis-(2-methoxy-4-nitro-5-sulfophenyl)-2H-tetrazolium-5-carboxanilide.

## Competing interests

The authors declare that they have no competing interests.

## Authors' contributions

JBP and ZG designed the MTT assay. BA, MML and MG checked its efficiency for a number of applications, 3 of which were presented in the paper. PS and PD supervised ZG and MML’s thesis, respectively, and drafted the manuscript. JBP supervised also ZG’s thesis. All authors read and approved the final manuscript.

## Supplementary Material

Additional file 1 **Time course of MTT reduction by *P. ramosa *germinated seeds.** Measurements 4 days post GR24 stimulation (3 nmol L^-1^) and after 0, 4, 8, 12, 24, and 32 hours of incubation with MTT. Scale bars =100 μm. A_570-630 nm_ was expressed as percentage of maximum absorbance at 32 h. Absorbance was measured after solubilization in the following conditions (22 h, 30°C and orbital shaking 150 rpm) (n = 8; bar = SE). Click here for file

Additonal file 2 **Formazan salt solubilization.** Solubilization from germinated seeds was assessed 4 days post G24 stimulation and after incubation with MTT for 24 hours at 21°C. Solubilization conditions: 21°C without shaking or 30°C under orbital shaking (150 rpm). A_570-630 nm_ was expressed as percentage of maximum absorbance measured after a solubilization period of 67 h at 21°C or 12 h at 30°C (n = 24; bar = SE). Click here for file
