# Supplementary figures and images for: A high-throughput seed germination assay for root parasitic plants
Source: Plant Methods. 2013 Aug 6;9:32. doi: 10.1186/1746-4811-9-32 (PMC3751143; doi:10.1186/1746-4811-9-32)

## Slide 1
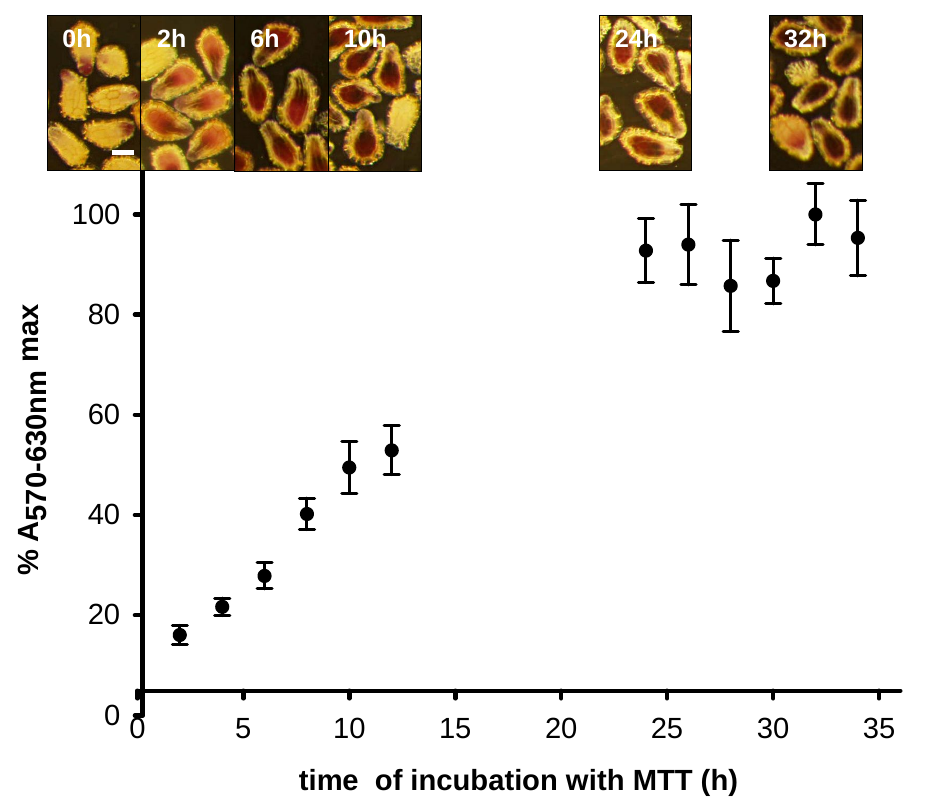

0h
2h
6h
10h
24h
32h

Supplement: Additional file 1 — Time course of MTT reduction by P. ramosa germinated seeds. Measurements 4 days post GR24 stimulation (3 nmol L-1) and after 0, 4, 8, 12, 24, and 32 hours of incubation with MTT. Scale bars =100 μm. A570-630 nm was expressed as percentage of maximum absorbance at 32 h. Absorbance was measured after solubilization in the following conditions (22 h, 30°C and orbital shaking 150 rpm) (n = 8; bar = SE). [file 1746-4811-9-32-S1.pptx]

## Slide 1
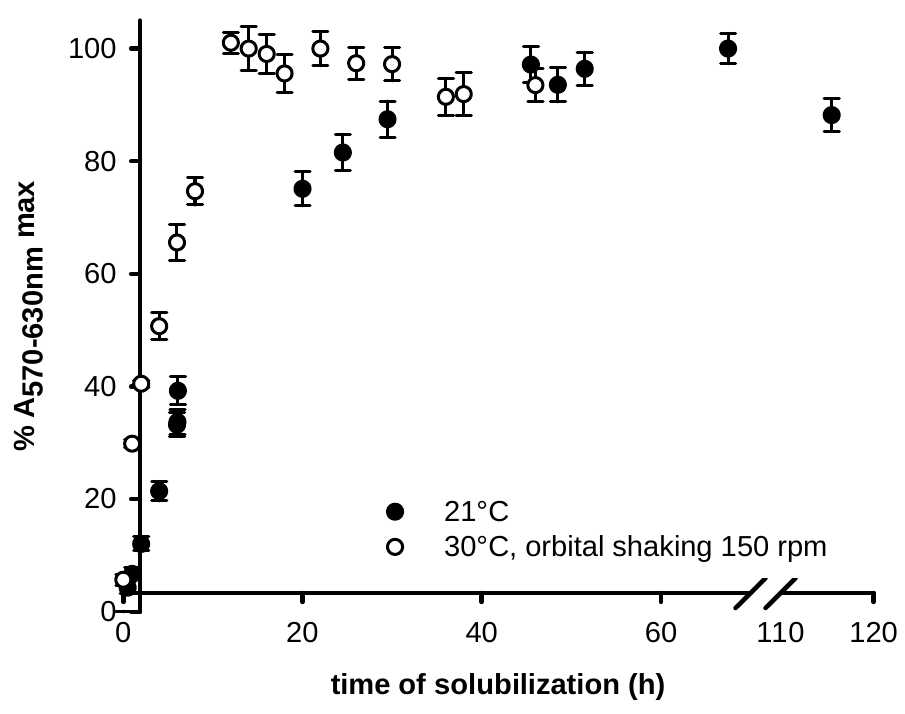

Supplement: Additonal file 2 — Formazan salt solubilization. Solubilization from germinated seeds was assessed 4 days post G24 stimulation and after incubation with MTT for 24 hours at 21°C. Solubilization conditions: 21°C without shaking or 30°C under orbital shaking (150 rpm). A570-630 nm was expressed as percentage of maximum absorbance measured after a solubilization period of 67 h at 21°C or 12 h at 30°C (n = 24; bar = SE). [file 1746-4811-9-32-S2.pptx]
